# Nonarteritic Anterior Ischemic Optic Neuropathy Induced Retinal Folds and Deformations

**DOI:** 10.1167/iovs.17-22140

**Published:** 2017-08

**Authors:** Mark J. Kupersmith, Patrick A. Sibony, Sarita Dave

**Affiliations:** 1 New York Eye and Ear Infirmary and Icahn School of Medicine at Mount Sinai, New York, New York, United States; 2 Department of Ophthalmology, State University of New York at Stony Brook, Stony Brook, New York, United States

**Keywords:** nonarteritic anterior ischemic optic neuropathy, NAION, OCT, peripapillary wrinkles, retinal folds, choroidal folds

## Abstract

**Purpose:**

We hypothesized that the edema/swelling in the retina due to acute nonarteritic anterior ischemic optic neuropathy (NAION) can induce retinal folds (RF). We determined the pattern and frequency of folds in NAION at presentation and in follow-up, and the relationship between folds and a number of functional and structural parameters over time.

**Methods:**

We prospectively studied eyes with acute NAION by spectral-domain optic coherence tomography (SD-OCT). We used transaxial and en face views to evaluate the presence of peripapillary fluid (PPF), peripapillary wrinkles (PPW), RF, choroidal folds (CF), creases, macular edema, and vitreous traction on the optic disc. Retinal deformations were correlated with the retinal nerve fiber layer (RNFL) thickness, logMAR visual acuity (VA) and mean deviation (MD).

**Results:**

At presentation, 60 eyes had mean RNFL = 224 ± 75 μm, no vitreous traction, and similar VA and MD regardless of the retinal deformation or macular edema. There was PPF in 73%, PPW in 57%, RF in 38%, creases in 20%, and macular edema in 18% of eyes, and no CF. Eyes with retinal deformations had significantly greater RNFL thickness (*P* < 0.026). At 1 to 2 months, 49 eyes had reduction of the RNFL (112 ± 40 μm, *P* = 0.001) and unchanged VA and MD that did not correlate with fewer eyes having PPF (15%, *P* = 0.001), PPW (10%, *P* = 0.001), RF (10%, *P* = 0.001), creases (17%), and macular edema (0%, *P* = 0.007).

**Conclusions:**

RF in NAION reflect stresses and strains due to extracellular fluid without increased pressure in the retrolaminar tissue and subarachnoid space, seen with papilledema. In NAION, the deformations and their resolution do not correlate with vision loss.

Acute optic nerve injury due to nonarteritic anterior ischemic optic neuropathy (NAION) typically causes significant visual field defects and reduced visual acuity (VA), which almost always is permanent. In recent years imaging methods, such as optical coherence tomography and scanning laser polarimetry, have been used extensively to document swelling and injury at the level of the optic nerve head (ONH) and peripapillary retinal nerve fiber layer (RNFL),^1–6^ and secondary retinal ganglion cell layer (GCL)^7,8^ thinning or atrophy. Given the purported ischemic pathophysiology, other imaging techniques have explored the blood flow or the vasculature in the region of the ONH and peripapillary retina. Clinical observations and older optical imaging reports have suggested that acute swelling of the ONH in NAION causes significant changes in the peripapillary retina. When the retinal edema extends to the macula, it may contribute a component of vision loss that is reversible.^9^ Outer retinal fluid also is found in 100% of eyes with experimentally-induced NAION.^10^

Wrinkles and folds in the retina are seen with other causes of ONH swelling, such as papilledema. These develop when one or more forces are applied to the retinal layers, which are compliant substrate. The fold patterns are determined by the structural geometry of the ONH and retinal tissues and loading force conditions. Examination of retinal folds (RF) in patients with papilledema due to intracranial hypertension has provided some insights into the biomechanical effects of intracranial hypertension and ONH swelling. We recently reported several types of retinal and choroidal folds (CF) in patients with papilledema due to idiopathic intracranial hypertension (IIH) and how these folds change over time and with therapy.^11,12^ These folds have several stereotypic patterns: (1) peripapillary wrinkles (PPW), consisting of closely spaced concentric or spiral folds within a half disc diameter of the ONH and confined to the retinal nerve fiber layer (RNFL); (2) horizontal or radially oriented RF greater than one half disc diameter from the optic disc and predominantly affecting the middle and inner retinal layers in the posterior pole that spared the outer retina and choroid; (3) choroidal folds (CF), which almost always are associated with overlying RF, and (4) peripapillary outer RF characterized by deeply furrowed “creases” (“high water marks”).^13^ The pattern of folds correlated with two structural determinates: PPW and outer RF were associated with the magnitude of disc edema (thickened RNFL and volume), whereas CF correlated with the degree of anterior displacement of the peripapillary tissues and lamina cribrosa. Inner RF correlated with disc edema and shape deformation. RF diminished with treatment that reduced ONH swelling, RNFL thickness, and cerebrospinal fluid pressure at 6 months.^12^ PPW and outer RF eventually resolved over a longer interval and CF persisted over a longer interval despite treatment.

Although the pathophysiology of ONH swelling in NAION and papilledema differs, the volumetric swelling of the ONH is a structural feature common to both. Comparison of the types and patterns of RF that occur in each might provide insights into the biomechanical distinctions between disc edema alone (in NAION) and elevated cerebrospinal pressure induced disc edema. The purpose of this study was to determine the pattern and frequency of folds in NAION at presentation and in follow-up, and to examine the relationship between folds and a number of functional and structural parameters over time. Given that NAION does not have increased pressure on the sclera or within the optic canal as occurs in papilledema, we hypothesized the retinal deformations in NAION would show differences from those seen with papilledema. Since ischemia of the optic nerve is considered the cause of NAION, we explored whether the edema in the macula would significantly affect the vision loss at presentation or outcome. Lastly, we evaluated whether vitreous traction on the optic disc is a common contributory factor for NAION.^14–17^

## Methods

Over a 3-year period (2014–2016), we prospectively studied eyes with new onset NAION, within 15 days of patient-reported vision loss, and at 1 to 2 months of follow-up. Each subject had complete clinical evaluation and standard automated threshold perimetry performed using the Humphrey Field Analyzer (Carl Zeiss Meditec, Inc., Dublin, CA, USA) with SITA 24-2 standard perimeter strategy using size III (expressed as mean deviation [MD] in decibels [dB]), and spectral-domain optic coherence tomography (SD-OCT) of the optic disc and macula regions using the protocol detailed below at each visit. VA, measured by Snellen charts, is reported as logMAR values. This research was conducted with New York Eye and Ear Infirmary institutional review board approval and adhered to the tenets of the Declaration of Helsinki.

The inclusion criteria were acute painless unilateral vision loss within 15 days of the presentation evaluation, unilateral optic disc edema with RNFL thicker than the 95% limit of the control database from the Cirrus OCT (Carl Zeiss Meditec, Inc.), visual field loss consistent with NAION, relative afferent pupillary defect unless the fellow eye was previously affected, and no toxic or systemic infectious or inflammatory cause suggested by history or blood tests (complete hemogram, C-reactive protein, sedimentation rate, syphilis serology) performed in all patients.

OCT imaging was performed following pupillary dilation. For this study we used the Cirrus SD-OCT (Carl Zeiss Meditec, Inc.) with laser scanned 6 × 6 mm area, capturing of a cube of data consisting of 200 A-scans from 200 linear B- scans for the optic nerve and macula regions. Five line 9 mm high definition raster scans were performed horizontally through the optic disc and vertically through the papillomacular region of the retina (to maximize the chance of seeing horizontally- or radially-oriented folds). At least two volume scans were performed for each region on each eye and only images centered on the optic disc or macula with signal strength scores 6 or greater were analyzed. The Carl Zeiss Meditec, Inc. average peripapillary RNFL thickness was calculated in micrometers (μm) from values at 256 points in the peripapillary circumference. Raster scans were used to evaluate intraretinal fluid and delineate the presence and type of folds. Volume scans also were used for en face imaging with the Advanced Visualization Analysis program for the Cirrus SD-OCT to view the retina at the internal limiting membrane, middle layers, and outer retina/retinal pigment epithelium levels to delineate the presence and type of fold.

We used inspection of all images to determine the presence or absence of each retinal deformity. We used the same features for each type of fold, PPW, RF, CF, and creases, we previously defined and used in the study of IIH associated papilledema.^11–13^ PPW are located within the RNFL and appear as tightly spaced circumferential undulations on the surface of the disc surface or within a half disc diameter from the disc margin. RF are periodic intraretinal undulations at a distance greater than a half disc diameter from the disc margin. CF are folds in the retina due to undulations in the RPE/Bruch's membrane (RPE/BM) layer. Creases typically are single and located in the peripapillary outer retina and usually associated with outer retinal fluid or a deep furrow involving the ellipsoid zone to the outer nuclear layer. We measured the peak to trough width for PPW in each eye. Peripapillary intra- and subretinal fluid (PPF) was assessed with the 5-line raster images of the ONH region. Macular edema was assessed with raster images and en face images of the macula region. Vitreous traction on the ONH was determined via the raster images through the ONH region.

A *t*-test, adjusted for the five comparisons, was used to assess the RNFL thickness for eyes with and without each type of fold. A Pearson χ^2^ test was used to determine the relationship between having edema in the retina (PPF) and the presence of each type of fold. Bootstrap analysis was used to assess the presence of macular edema and the VA and MD. The RNFL thickness, VA and MD at baseline and at the 1-to 2-month exam were compared using the paired *t*-test.

## Results

The study included 59 subjects with acute NAION in 60 eyes, with one patient having consecutive NAION with the second eye affected 2 months after the first. No eyes were excluded from analysis due to missing images or artifacts that interfered with assessment. The average age was 61.2 ± 12.6 years (61.2 ± 13.1 years for 49 patients with follow-up evaluations) and there were 34 men and 25 women. A total of 12 patients had had NAION previously in the fellow eye. At presentation, the VA and MD were typical of NAION (Table). The RNFL thickness reflected the wide range of swelling of the optic disc.

**Table:**
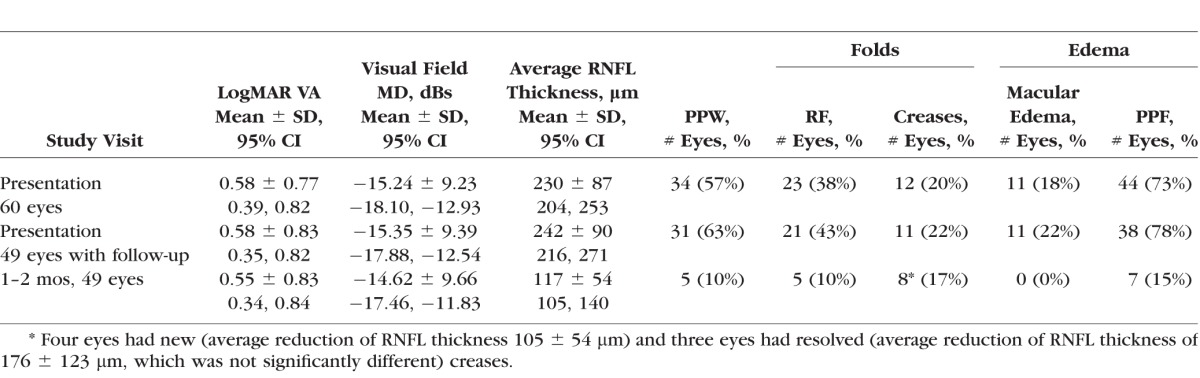
Affected Eye Vision and OCT Findings

**Figure 1:**
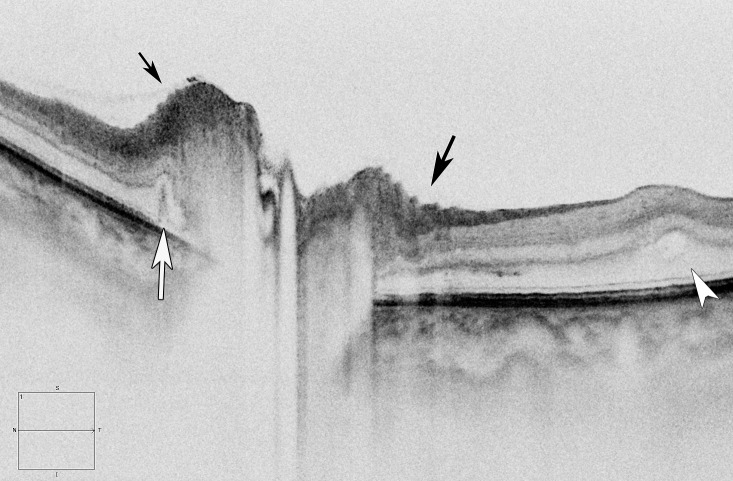
High definition horizontal raster line image through the ONH of a left eye with NAION at presentation shows an outer retina edema or fluid extending under the macula (white arrowhead) as well as PPW (black arrows) and a crease (white arrow).

**Figure 2:**
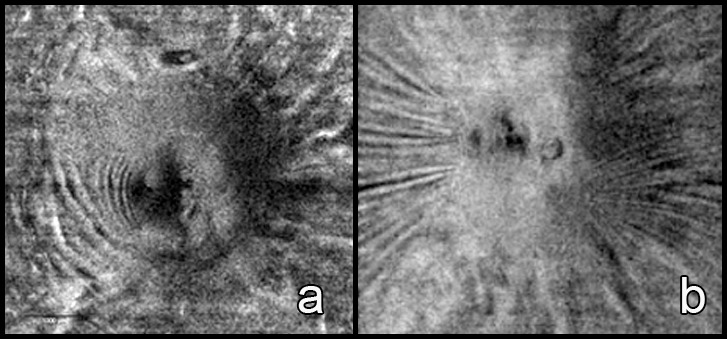
En face images of the ONH at the inner limiting layer level shows NAION at presentation (a) and papilledema (b) in right eyes. The NAION eye shows concentric RF expanding temporally in contrast to the radial RF with papilledema.

At presentation, the frequency of retinal deformations was highest for PPF, followed by PPW, then RF, then macular edema, then creases (Table; Figs. 1, 2). No CF were seen. Vitreous attached to the optic disc was identified commonly but no eyes had vitreal traction of the optic disc. PPF was located in the outer retina in 38 eyes (63%) and in the middle/inner retina in 12 (20%, six of which also were in the outer retina). Some type of fold or crease or PPF was found in 49 eyes (82%). All but one eye with macular edema had RF. The average PPW width, 137 ± 44 μm (95% confidence interval [CI], 117–160). The RF had concentric orientation only. The VA and MD were similar for NAION eyes with or without PPF or macular edema. The RNFL thickness was increased in eyes with PPF (246 ± 72 vs. 156 ± 31 μm, *P* = 0.005), PPW (265 ± 68 vs. 173 ± 48 μm, *P* = 0.02), RF (258 ± 77 vs. 200 ± 64 μm, *P* = 0.025), but not with macular edema (274 ± 56 vs. 213 ± 78 μm, *P* = 0.13) or creases (234 ± 29 vs. 228 ± 12 μm, *P* = 0.08). The number of all RF correlated (*r* = 0.65, *P* = 0.01) with the RNFL thickness at presentation. The χ^2^ values were significant for PPF and PPW (5.40, *P* = 0.017; odds ratio [OR], 4.25) and PPF and RF (9.50, *P* = 0.002; OR, 15.00) but not for PPF and creases (*P* = 0.11) or PPF and macular edema (*P* = 0.11).

**Figure 3:**
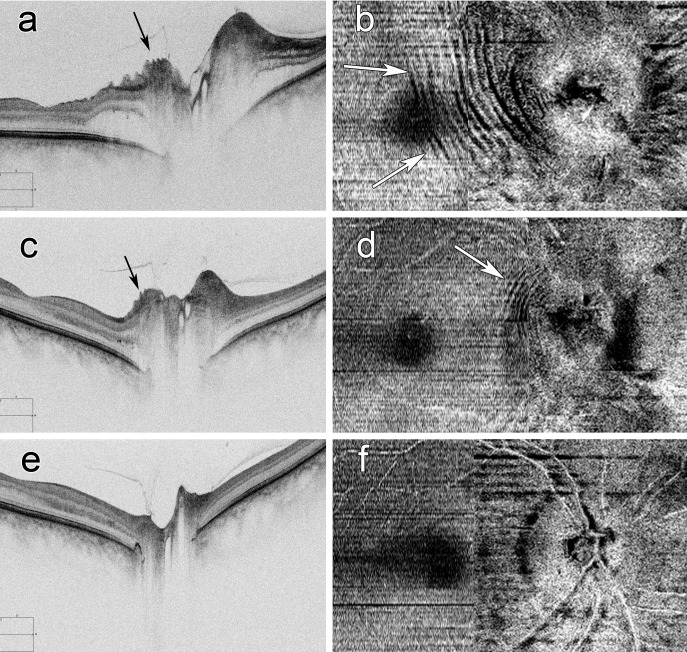
Images of a right eye with NAION: high definition raster images are on the left and en face images of macula and ONH region volume scans are on the right. The top images are at presentation (RNFL 329 μm; [a, b]), at 3 weeks in the middle (RNFL 201 μm; [c, d]) and at 7 weeks at the bottom (RNFL 71 μm; [e, f]). Outer retinal fluid in the peripapillary space around the ONH (a) is decreased at 3 weeks (c) and resolved at 7 weeks. PPW (black arrows) and concentric RF (white arrows) present at presentation are still present at 3 weeks but have resolved at 7 weeks. Note the concentric RF extend to the macula at presentation.

**Figure 4:**
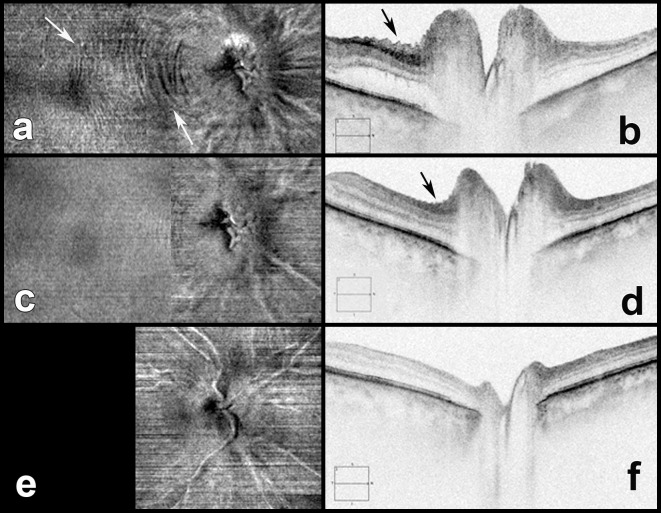
Images of a right eye with NAION: en face images of macula and ONH region volume scans are on the left and high definition raster images are on the right. The top images are at presentation (RNFL 334 μm; [a, b]), at 5 days in the middle (RNFL 277 μm; [c, d]) and at 2 months at the bottom (RNFL 85 μm; [e, f]). Outer retinal fluid in the peripapillary space around the ONH and concentric RF (white arrows) extend to the macula (a, b) and are resolved at 5 days (c, d). This suggests the concentric pattern of RF is due to retinal edema. PPW (black arrows) still are seen at 5 days but not at 2 months.

At the follow-up evaluation at 1 to 2 months, in 49 eyes the OCT findings (Figs. 3, 4) were significantly changed from the baseline for all assessments except the creases (Table). The RNFL thickness was reduced (*P* = 0.001) and there were fewer eyes with PPF (*P* = 0.001), PPW (*P* = 0.001), RF (*P* = 0.001), and macular edema (*P* = 0.007). Four eyes had new creases, but no eyes had new PPF or macular edema, PPF, or any other type of fold. There was no significant difference for VA or MD at 1 to 2 months compared to the same eye at presentation. Five eyes (10%) improved the VA by two lines or more and nine (18%) improved the MD by more than 3 dBs. Three eyes (6%) worsened the VA by two lines or more and nine (18%) had worse MD by more than 3 dBs.

At the follow-up evaluation, the VA and MD were similar for the eyes that had resolution of macular edema and for eyes that had a normal macula at baseline. There was a nonsignificant trend for eyes with macular edema at presentation to improve the VA (−0.21 ± 1.38; 95% CI, −1.17–0.67) more than for eyes with a normal macula at presentation (0.004 ± 0.44; 95% CI, −0.12–0.16). The change in MD was similar for eyes in both groups (0.34 ± 6.50 dB; 95% CI, −3.85–4.44 for eyes with macular edema at presentation and 2.16 ± 8.11 dB; 95% CI, −0.41–4.91 for eyes with normal macula at presentation).

Unplanned evaluations were performed on 11 eyes within 2 weeks of presentation. Peripapillary retina fluid resolved in five of the nine eyes with fluid at presentation, one of which resolved in less than 1 week (Fig. 3). PPW resolved in three of the eight eyes with PPW at presentation. RF resolved in five of the seven eyes with RF at presentation. Creases resolved in two of three eyes with creases at presentation and one eye had a new crease.

## Discussion

RF, particularly PPW and RF, and retinal edema were common but CF did not occur at presentation of NAION. The RF and retinal edema frequently abated or were significantly less within 1 to 2 months of onset. RF of all types was seen in 82% of eyes with acute NAION, which is similar to the frequency of 73% in eyes with untreated papilledema due to IIH.^11^ Folds in the retina are a common response to stresses and strain caused by the increased pressure exerted on the posterior globe and tissues of the optic canal as well as from ONH swelling/volume expansion and edema extending into the peripapillary retina. NAION and papilledema associated with IIH^11,13^ have PPW, RF, and outer retinal creases.

There are fundamental differences between the mechanical forces and pathologic mechanisms that cause these ONH and retina deformations, which account for the different types and patterns of folds seen in NAION and papilledema. With respect to the RF, we speculated that NAION only demonstrates a concentric configuration in NAION due to the mechanical effects of middle and outer retina fluid/edema (PPF) characteristically seen in eyes with NAION. Additionally, the RF disappear as the PPF resolves. In contrast, the RF in papilledema often have a radial, and less commonly horizontal, pattern. Other features in the pattern of folds (such as no CF and narrower PPW width in NAION) in NAION further distinguish NAION from the folds seen in papilledema. First, the time course is rapid for the onset and then swelling reduction in NAION (notwithstanding case reports suggesting ONH swelling can be present for weeks before vision loss^18.19^), and slower and insidious, typically over weeks to months before diagnosis and to time of improvement in many patients with papilledema. Second, the increased pressure in the intracranial or subarachnoid perioptic space, and at the scleral flange, which typically occurs with papilledema, is absent in patients with NAION and is likely the explanation for not having CF or radial-oriented RF in NAION eyes. Third, NAION causes immediate tissue injury and loss of integrity of ONH axons so the response to local tissue pressure strains will be different from ONH swelling with intact cytoarchitecture. Fourth, the amount of extracellular fluid from damage axons and endothelium may be higher in NAION.

The amount of swelling as measured by increased RNFL thickness was greater in NAION eyes that had PPF, PPW, and RF. In contrast, for papilledema (data from 125 study eyes of subjects average age 33 ± 7 years^20,21^) increased RNFL thickness (280 ± 172 μm; 95% CI, 245–315, which was similar in NAION eyes) was greater only in eyes with RF (*P* = 0.003) and macular edema (*P* = 0.045). In NAION, the two most common folds, PPW and RF, were significantly more common when retinal edema was present. It would be of interest to determine whether the amount of edema could be correlated with the number of these folds.

The ONH swelling of NAION typically improves over 1 month and resolves within 3 months while papilledema due to IIH can last for many months or years unless the cerebrospinal fluid pressure or retrobulbar subarachnoid pressure is markedly reduced. It is noteworthy that all the retinal deformations seen in NAION and only the RF of IIH showed significant improvement over the first months as the RNFL swelling and thickening subsided.

PPW in NAION also showed different behavior than those seen in papilledema due to IIH, including being wider (137 μm for NAION and 103 ± 13 μm; 95% CI, 96–110 for IIH). PPW, which are seen in papilledema and NAION at presentation, did not have a delayed onset in NAION as we observed in papilledema.^12^ In the latter disorder axons are stretched more gradually by the ONH swelling but frequently remain intact and as the swelling recedes, stretched axons fold back on themselves. The resulting PPW can be seen as “Paton's folds” on ophthalmoscopy.^13^ In NAION, early injury to the optic nerve axons (as demonstrated by loss of birefringence^6^) that reduces the elasticity and the rapid axonal loss account for the lack of delayed PPW. This also suggests that optic nerve axons in eyes with papilledema maintain elasticity better than in NAION eyes.

In a retrospective record review using time domain OCT, Hedges et al.^9^ reported that of 76 NAION eyes, eight (11%) had outer retinal fluid in the macula. This rate is similar to our 18% frequency of macular edema seen at presentation despite the use of different methodologies for imaging. At presentation, we found eyes with macular edema did not have worse VA or MD. Also, there was only a nonsignificant trend for VA to improve as the macula normalized. Although seven of the eight eyes in the report of Hedges et al.^9^ showed improved VA and/or the visual field as the retinal fluid resolved, several of the cases had only minor improvement compatible with the natural history of all NAION. However, several cases had mild visual field MD reduction at presentation that did not seem compatible with the severe VA. This suggests that one of the visual performance measures might not be as accurate as when these data are collected prospectively by an investigator using rigorous assessment. Also, cases were included with OCT images collected at up to 4 weeks of vision loss, a time point our data shows resolution of most retinal fluid and deformations.

There has been a longstanding speculation about the role of vitreous traction in the pathogenesis of NAION.^15–17^ A careful examination of the vitreous attachments to the disc on the high definition raster images failed to demonstrate evidence of vitreous traction or partial detachment in this cohort of patients.

RF are found commonly at presentation in eyes with NAION and papilledema. The concentric pattern of RF and lack of CF reflect the different biomechanical factors in NAION. We anticipate that the presence of the various types and patterns of RF will be useful to distinguish ONH swelling/edema due to acquired diseases from development or congenital causes of optic disc elevation, such as drusen, even when the RNFL appears markedly thickened.

## References

[1] Danish-Meyer H, Carroll S, Ku J, Correlation of retinal nerve fiber layer measured by scanning laser polarimeter to visual field in ischemic optic neuropathy. *Arch Ophthalmol*. 2006; 124: 172– 1726. 10.1001/archopht.124.12.1720 17159031

[2] Contrearas I, Noval S, Rebolleda G, Munoz-Negrete F. Follow-up of nonarteritic anterior ischemic optic neuropathy with optical coherence tomography. *Ophthalmology*. 2007; 114: 2338– 2344. 17719640 10.1016/j.ophtha.2007.05.042

[3] Hood D, Anderson S, Rouleau J, Retinal nerve fiber structure versus visual field function in patients with ischemic optic neuropathy. *Ophthalmology*. 2008; 115; 904– 910. 17870170 10.1016/j.ophtha.2007.06.001 PMC2987576

[4] DeLeon-Ortega J, Carrol K, Arthur S, Girkin C. Correlations between retinal nerve fiber layer and visual field in eyes with nonarteritic anterior ischemic optic neuropathy. *Am J Ophthalmol*. 2007; 143: 288– 294. 17157797 10.1016/j.ajo.2006.09.045 PMC1906588

[5] Bellusci C, Savini G, Carbonelli M, Carelli V, Sadaun A, Barboni P. Retinal nerve fiber layer thickness in nonarteritic anterior ischemic optic neuropathy: OCT characterization of the acute and resolving phases. *Graefs Arch Clin Exp Ophthalmol*. 2008; 246: 641– 647. 10.1007/s00417-008-0767-x 18305953

[6] Kupersmith M, Anderson S, Durbin M, Kardon R. Scanning laser polarimetry, but not optical coherence tomography predicts permanent visual field loss in nonarteritic anterior ischemic optic neuropathy. *Invest Ophthalmol Vis Sci*. 2013; 54: 5414– 5419. 10.1167/iovs.13-12253 PMC3747791 23838768

[7] Aggarwal D, Tan O, Huang D, Sadun A. Patterns of ganglion cell complex and nerve fiber layer loss in nonarteritic ischemic optic neuropathy by fourier-domain optical coherence tomography. *Invest Ophthalmol Vis Sci*. 2012; 53: 4539– 4545. 22678499 10.1167/iovs.11-9300 PMC4625826

[8] Kupersmith M, Garvin M, Wang JK, Durbin M, Kardon R. Retinal ganglion cell layer thinning within one month of presentation for nonarteritic anterior ischemic optic neuropathy. *Invest Ophthalmol Vis Sci*. 2016; 57: 3588– 3593. 27388052 10.1167/iovs.15-18736 PMC5996873

[9] Hedges TR III, Vuong L, Gonzalez-Garcia A, Mendoza-Santiestban C, Amaro-Quierza M. Subretinal fluid from anterior ischemic optic neuropathy by optical coherence tomography. *Arch Ophthalmol*. 2008; 126: 812– 815. 18541844 10.1001/archopht.126.6.812

[10] Yu C, Ho JK, Liao Y. Subretinal fluid is common in experimental nonarteritic anterior ischemic optic neuropathy. *Eye*. 2014; 28: 1494– 1501. 25257770 10.1038/eye.2014.220 PMC4268460

[11] Sibony P, Kupersmith MJ, Feldon S, Wang JK, Garvin M, and the OCT Substudy Group for the NORDIC Idiopathic Intracranial Hypertension Treatment Trial. Retinal and choroidal folds in papilledema. *Invest Ophthalmol Vis Sci*. 2015; 56: 670– 680. 10.1167/iovs.15-17459 PMC4562343 26335066

[12] Kupersmith M, Sibony P, Feldon S, The effect of treatment of idiopathic intracranial hypertension on prevalence of retinal and choroidal folds. *Am J Ophthalmol*. 2017; 176: 77– 86. 28040526 10.1016/j.ajo.2016.12.017 PMC5376523

[13] Sibony P, Kupersmith M “Paton's Folds” revisited: peripapillary wrinkles, folds, and creases in papilledema. *Ophthalmology*. 2016; 123: 1397– 1399. 26778344 10.1016/j.ophtha.2015.12.017 PMC4877233

[14] Hedges TR, Flattem NL, Bagga A. Vitreopapillary traction confirmed by optical coherence tomography. *Arch Ophthalmol*. 2006; 124: 279– 281. 16476902 10.1001/archopht.124.2.279

[15] Modarres M, Sanjar MS, Falavarjani KG Vitrectomy and release of presumed epipapillary vitreous traction for treatment of nonarteritic anterior ischemic optic neuropathy associated with partial posterior vitreous detachment. *Ophthalmology*. 2007; 114: 340– 344. 17270682 10.1016/j.ophtha.2006.07.063

[16] Parsa CF, Hoyt W. Nonarteritic anterior ischemic optic neuropathy (NAION): a misnomer. Rearranging pieces of a puzzle to reveal a nonischemic papillopathy caused by vitreous separation. *Ophthalmology*. 2015; 122: 439– 442. 25703466 10.1016/j.ophtha.2014.11.011

[17] Shen B, Macintosh P. Posterior vitreous detachment associated with non-arteritic ischaemic optic neuropathy. *Neuro-Ophthalmology*. 2016; 40: 234– 236. 27928412 10.1080/01658107.2016.1206575 PMC5123113

[18] Gordon R, Burde R, Slamovits T. Asymptomatic optic disc edema. *J Neuroophthalmol*. 1997; 17: 29– 32. 9093957

[19] Subramanian P, Gordon L, Bonelli L, Arnold A. Progression of asymptomatic optic disc swelling to non-arteritic anterior ischaemic optic neuropathy. *Br J Ophthalmol*. 2017; 101: 671– 675. 27565987 10.1136/bjophthalmol-2016-309250

[20] OCT Sub-Study Committee for the NORDIC Idiopathic Intracranial Hypertension Study Group. Baseline OCT measurements in the Idiopathic Intracranial Hypertension Treatment Trial: Part I. Quality control, comparisons and variability. *Invest Ophthalmol Vis Sci*, 2014; 55: 8173– 8179. 25370510 10.1167/iovs.14-14960 PMC4266084

[21] OCT Sub-Study Committee for the NORDIC Idiopathic Intracranial Hypertension Study Group. Baseline OCT measurements in the Idiopathic Intracranial Hypertension Treatment Trial: Part II. Correlations and relationship to clinical features. *Invest Ophthalmol Vis Sci*. 2014; 55: 8180– 8188. 25370510

